# Analyses of Mitogenome Sequences Revealed that Asian Citrus Psyllids (*Diaphorina citri*) from California Were Related to Those from Florida

**DOI:** 10.1038/s41598-017-10713-3

**Published:** 2017-08-31

**Authors:** Fengnian Wu, Luci Kumagai, Yijing Cen, Jianchi Chen, Christopher M. Wallis, MaryLou Polek, Hongyan Jiang, Zheng Zheng, Guangwen Liang, Xiaoling Deng

**Affiliations:** 1 Guangdong Province Key Laboratory of Microbial Signals and Disease Control/Laboratory of Insect Ecology, College of Agriculture, South China Agricultural University, Guangzhou, Guangdong, China; 2 California Department of Food and Agriculture, Plant Pest Diagnostic Center, Sacramento, California, USA; 3 United States Department of Agriculture-Agricultural Research Service, San Joaquin Valley Agricultural Sciences Center, Parlier, California, USA; 4 National Clonal Germplasm Repository for Citrus and Dates, Riverside, California, USA

## Abstract

Asian citrus psyllid (ACP, *Diaphorina citri* Kuwayama) transmits “*Candidatus* Liberibacter asiaticus” (CLas), an unculturable alpha-proteobacterium associated with citrus Huanglongbing (HLB). CLas has recently been found in California. Understanding ACP population diversity is necessary for HLB regulatory practices aimed at reducing CLas spread. In this study, two circular ACP mitogenome sequences from California (mt-CApsy, ~15,027 bp) and Florida (mt-FLpsy, ~15,012 bp), USA, were acquired. Each mitogenome contained 13 protein coding genes, 2 ribosomal RNA and 22 transfer RNA genes, and a control region varying in sizes. The Californian mt-CApsy was identical to the Floridian mt-FLpsy, but different from the mitogenome (mt-GDpsy) of Guangdong, China, in 50 single nucleotide polymorphisms (SNPs). Further analyses were performed on sequences in *cox1* and *trnAsn* regions with 100 ACPs, SNPs in *nad1*-*nad4*-*nad5* locus through PCR with 252 ACP samples. All results showed the presence of a Chinese ACP cluster (CAC) and an American ACP cluster (AAC). We proposed that ACP in California was likely not introduced from China based on our current ACP collection but somewhere in America. However, more studies with ACP samples from around the world are needed. ACP mitogenome sequence analyses will facilitate ACP population research.

## Introduction

The Asian citrus psyllid [ACP, *Diaphorina citri* Kuwayama (Hemiptera: Liviidae)] is an important pest of citrus trees. ACP transmits an unculturable alpha-proteobacterium, “*Candidatus* Liberibacter asiaticus” (CLas) which is associated with citrus Huanglongbing (HLB, yellow shoot disease, also called citrus greening disease). HLB currently is threatening citrus production worldwide^1^. There is currently no cure for HLB. The disease is managed mainly through the use of HLB-free nursery stocks and prevention of CLas infection and spread in the field. Therefore, control of ACP is a critical component of HLB regulatory practices designed to prevent possible CLas spread, and its success depends on comprehensive knowledge of the insect.

The first description of ACP was from Taiwan in 1908^2^, one of the four regions (Guangdong, Guangxi, Fujian and Taiwan) in southern China known for its long history of HLB^3^. The geographical origin of ACP was considered to be Southwest Asia such as India^4, 5^. While ACP was not known to transmit HLB pathogens, Chen demonstrated the association of a biological factor associated with HLB development in a four-year experiment^6^. Lin further proved the involvement of insects in HLB by an insecticide-spraying experiment^7^. Later, ACP was shown to transmit HLB pathogens in India, the Philippines^8, 9^ and Guangdong, China^10^.

In the United States, ACP was first found in Palm Beach County, Florida in 1998 and later on in other parts of the southeastern USA^11, 12^. ACP was first detected in southern California (San Diego County) in 2008^13^ and continues spreading northward. We have research interests in tracking HLB development in California and demonstrated that the Californian strains of CLas were associated with Asiatic strains but not the Floridian strain^14^. Yet, the possible origin of the Californian ACP has not been studied.

ACP was originally described based on morphological traits^2^. This psyllid species was separated from other psyllid species based on the pattern of black-brown maculation in the forewings, the wing venation, and genitalic structures^2^. Over the years, there have been several reclassification efforts in placing the species in different psylloid families, and currently it is a member of the Liviidae^15^. However, to our knowledge, there has not been a morphological description of ACP variation under the species level. To study the ACP population from different geographical regions, the partial cytochrome oxidase 1 (*cox1*) gene in the mitogenome of ACP has been used^16–20^. De León *et al*. found that two separate introductions or founding events of ACP occurred in the Americas, one in South America and one in North America^20^. Boykin *et al*. identified eight ACP haplotypes based on single nucleotide polymorphisms (SNPs) of 212 ACP individuals from 52 collections and suggested two major ACP groups between southwestern and southeastern Asia. The ACP that recently invaded Florida, USA, and Mexico belonged to the southwestern Asia group^16^.

This study attempts to search for the origin(s) of the Californian ACP as well as provide more gene access information of ACP mitogenomes. To this end, the mitogenomes of ACP from California and Florida were sequenced, and compared with a previously reported complete ACP mitogenome from Guangdong, China (mt-GDpsy, NC_030214)^21^. The findings of this study improve current understanding of ACP population diversity and have implications on HLB regulatory practices.

## Results

### The complete mitogenome sequences of ACP from California and Florida

**Figure 1:**
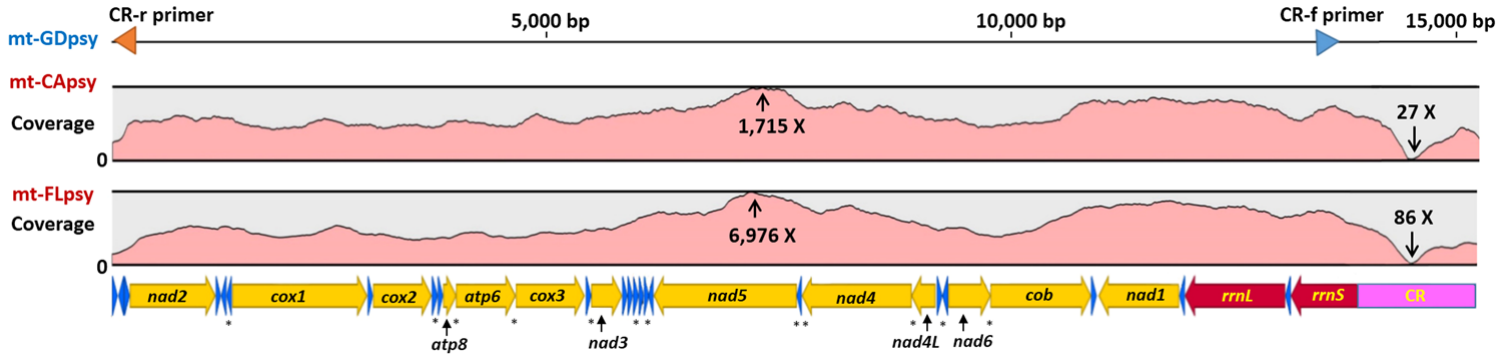
A schematic representation of mitogenome sequences of *Diaphorina citri* from California (mt-CApsy) and Florida (mt-FLpsy) in referencing to mitogenome sequence from Guangdong, China (mt-GDpsy). In the top line, the orange and blue arrows represent the forward and reversed primers (CR-r and CR-f) used to verify *D. citri* mitogenome circularity by PCR. Note in the coverage graphs of both mt-CApsy and mt-FLpsy, the highest is in *nad5* and the lowest in control region (CR). Majority strand (J-strand) is indicated by forward arrows, and minority strand (N-strand) in the opposite direction. *atp* = ATP synthase, *cob* = cytochrome oxidase b, *cox* = cytochrome oxidase c, *nad* = NADH dehydrogenase subunits, *rrnS* = small ribosomal RNA subunit and *rrnL* = large ribosomal RNA subunit. A star “*” indicates the location of a gene overlap. Color codes: orange = protein coding genes, blue = tRNA genes, red = rRNA genes, and pink = CR.

For the Californian ACP, 4.09 × 10^7^ reads (251 bp per read) were generated from MiSeq sequencing. A *de novo* assembling generated 85,803 contigs (≥1,000 bp). One contig of 15,013 bp was identified by referencing to mt-GDpsy. The mitogenome was enclosed by PCR, sequenced, designated as mt-CApsy (15,027 bp). The average coverage was 1,012 X (ranging from 27 to 1,715 X) using 60,824 reads (Fig. 1).

For the Floridian ACP, 5.46 × 10^7^ reads (301 bp per read) were generated. Referenced to mt-GDpsy, a contig of 14,989 bp was identified. Similar to mt-CApsy, the mitogenome was enclosed and designated as mt-FLpsy with the size of 15,012 bp and an average coverage of 3,843 X (ranging from 86 to 6,976) using 193,283 reads. The highest coverages in both mitogenomes were located in *nad5* genes, and the lowest coverages were in control regions (CRs) (Fig. 1).

Sequence annotation showed that both mitogenomes included the entire set of 37 genes usually present in animal mitogenomes^22^, including 13 protein-coding genes (PCGs), 22 transfer RNA (tRNA) genes, two ribosomal RNA (rRNA) genes, and an A + T-rich CR (Fig. 1). The two mitogenomes had the same gene order, gene direction and start/stop codons of each PCGs as that of mt-GDpsy. Twenty-three genes were on the majority strand (J-strand), and the other 14 genes on the minority strand (N-strand) (Fig. 1). Sizes of all intergenic regions ranged from 2 to 21 bp with the exceptions of CR. Gene overlaps were also observed: four between PCGs (1–7 bp), five between tRNA genes (1–11 bp), and three between PCG and tRNA genes (1–3 bp) (Fig. 1). In tRNA gene comparisons, the lengths of *trnAsn* of mt-CApsy and mt-FLpsy (66 bp) were 2 bp shorter than mt-GDpsy (68 bp), yet the lengths of *rrnL* of mt-CApsy and mt-FLpsy (1,136 bp) were both 2 bp longer than mt-GDpsy (1,134 bp).

**Figure 2:**
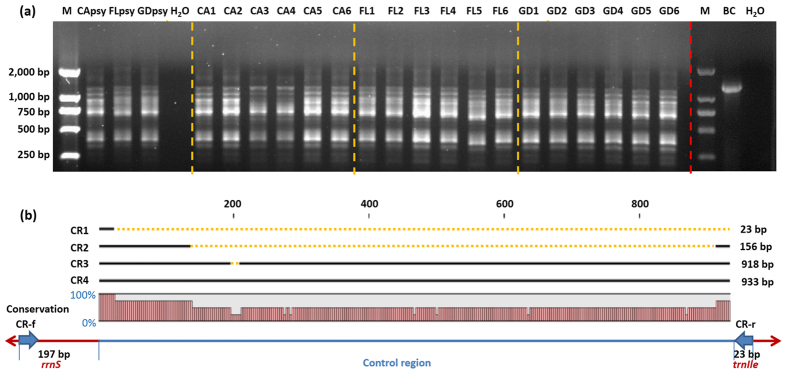
PCR and schematic nucleotide sequence alignments of the control region (CR) of *Diaphorina citri*. (**a**) Multiple amplicons of PCR of CR of individual *D. citri* from California, Florida and Guangdong-China based on primer set CR-f/CR-r. M, DNA Marker (2,000 bp); CApsy, FLpsy and GDpsy, *D. citri* from California, Florida and Guangdong-China, respectively, used for mitogenome sequencing; CA1-6: *D. citri* individuals from southern California, USA; FL1-6: *D. citri* individuals from Immokalee, Florida, USA; GD1-6: *D. citri* individuals from Guangzhou, Guangdong, China. BC, *Bactericera cockerelli*, showing a single amplicon from its CR as published previously^24^. (**b**) Schematic nucleotide sequence alignments of four variable amplicon clones from CR in a *D. citri* individual from California. *rrnS* = small ribosomal RNA.

As shown in Fig. 2a, instead of one single band such as other psyllids^23–26^, PCR with the designed CR primers yielded multiple DNA bands, typically two strong bands with weaker bands of different sizes from ACP. The same multiple band patterns were observed from additional sets of six psyllids each from California, Florida, and Guangdong (Fig. 2a). The cloned DNA bands of mt-CApsy and mt-FLpsy were 1,153 and 1,138 bp, corresponding to the CR length of 933 bp and 918 bp, respectively. In addition, three other DNA amplicons (243, 376 and 1,138 bp) were also cloned and sequenced from Californian ACPs. The size of each CR was 23, 156 and 918 bp, respectively (Fig. 2b).

### Nearly complete mitogenome sequences

Due to the high cost of MiSeq sequencing, a PCR-Sanger sequencing method was developed and used to acquire a total of 30 more nearly-complete mitogenome sequences, ten from Los Angeles County of California, ten from City of Immokalee of Florida, and ten from Guangzhou City of Guangdong (see Supplementary Tables S1 and S2). The nearly-complete mitogenome sequences lacked of complete CRs. All sequences had the lengths of 14,094 bp. All ten nearly-complete mitogenome sequences from the same region showed 100% matches to each of the correspondent complete mitogenome sequences, i.e. mt-CApsy (California), mt-FLpsy (Florida) and mt-GDpsy (Guangdong).

### Phylogenetic analyses based on 37 mitochondrial genes in complete mitogenomes

**Figure 3:**
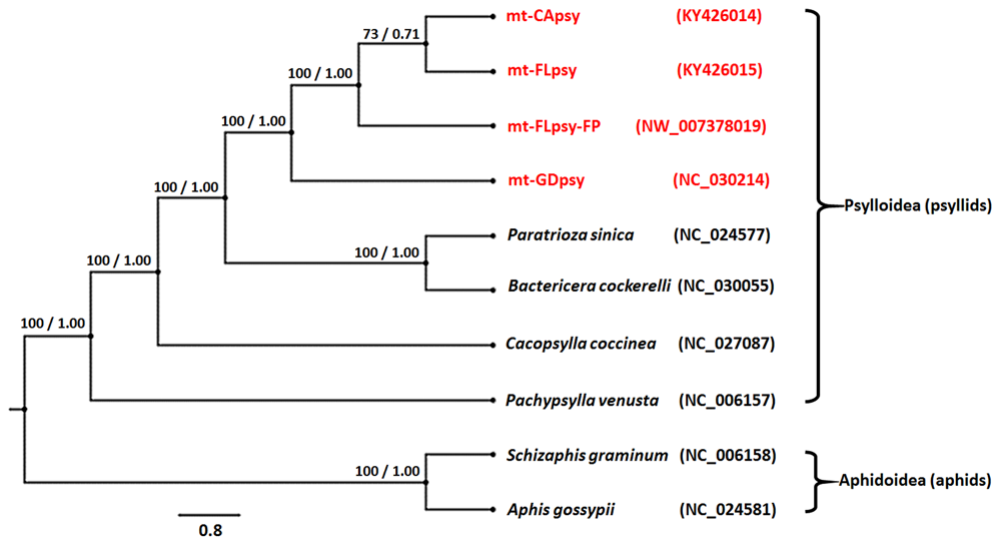
Phylogenetic analyses of psyllids based on complete mitogenome sequences excluding control regions. Numbers at the nodes are bootstrap values of maximum likelihood method/posterior probabilities of Bayesian inference method. *Diaphorina citri* was highlighted with “red”. The mitogenomes of *Schizaphis graminum* and *Aphis gossypii* were used as outgroup. Numbers in the brackets represent the GenBank accession numbers.

With both maximum likelihood (ML) and Bayesian inference (BI) methods, mt-CApsy, mt-FLpsy, mt-FLpsy-FP and mt-GDpsy formed a unique cluster within Psylloidea Superfamily that included *Cacopsylla coccinea* (Kuwayama), *Paratrioza sinica* (Yang & Li) [syn. *Bactericera gobica* (Losinova)], *Bactericera cockerelli* (Šulc) and *Pachypsylla venusta* (Osten-Sacken)^2, 23–26^ (Fig. 3). Sequence of mt-FLpsy-FP was an incomplete Floridian ACP mitogenome (14,094 bp) deposited in GenBank (NW_007378019, 169,790 bp) by Reese *et al*. in 2013. The high bootstrap value (100%) in ML analyses and Bayesian posterior probabilities (1.00) in BI analyses indicated the high stability of the ACP cluster. Yet, within the ACP cluster, mt-CApsy was more related to mt-FLpsy and mt-FLpsy-FP than to mt-GDpsy (Fig. 3).

### SNPs among 37 mitochondrial genes in ACP mitogenomes

**Figure 4:**
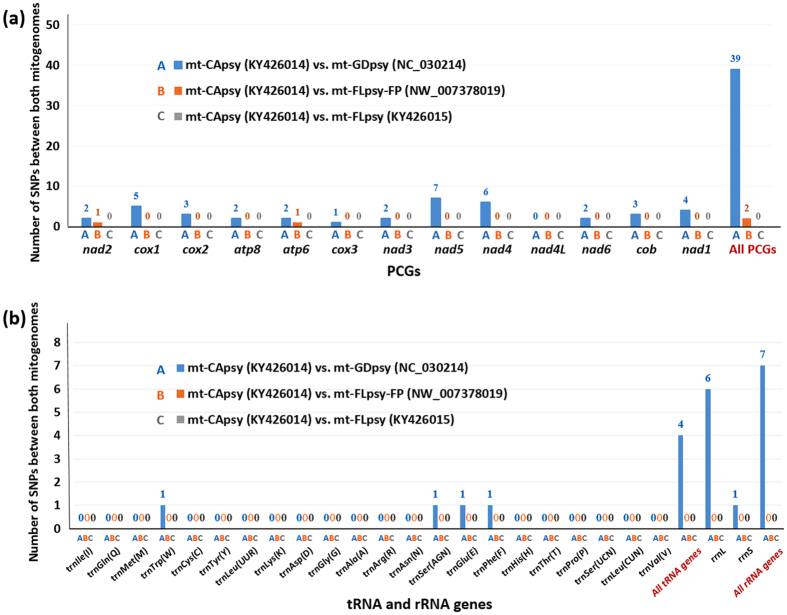
Pair-wise numbers of single nucleotide polymorphisms (SNPs) of (**a**) mitochondrial protein coding genes (PCGs), (**b**) transfer RNA (tRNA) genes and ribosomal RNA (rRNA) genes among three *Diaphorina citri* mitogenomes from California, Florida and Guangdong, China. Numbers in the brackets represent the GenBank accession numbers.

Numbers of SNPs in each gene among the three mitogenomes were listed in Fig. 4. There was no SNP found between mt-CApsy and mt-FLpsy (column C in Fig. 4a,b). Two SNPs in *nad2* and *atp6* were present between mt-CApsy and mt-FLpsy-FP. However, confirmations of the two SNPs were not successful with 20 ACP individuals collected from Immokalee, Florida. Fifty SNPs were found between mt-CApsy and mt-GDpsy (column A in Fig. 4a,b). Among them, 39 SNPs were in PCGs, four in tRNA genes (*trnTrp*, *trnSer* ^AGN^, *trnGlu* and *trnPhe*) and seven in rRNA genes (6 in *rrnL* and 1 in *rrnS*). Among all the PCGs, *nad5*, *nad4* and *cox1* had the most number of SNPs (7, 6 and 5 respectively), and no SNPs were found in *nad4L* (Fig. 4a).

### Phylogenetic analyses of *cox1*

**Figure 5:**
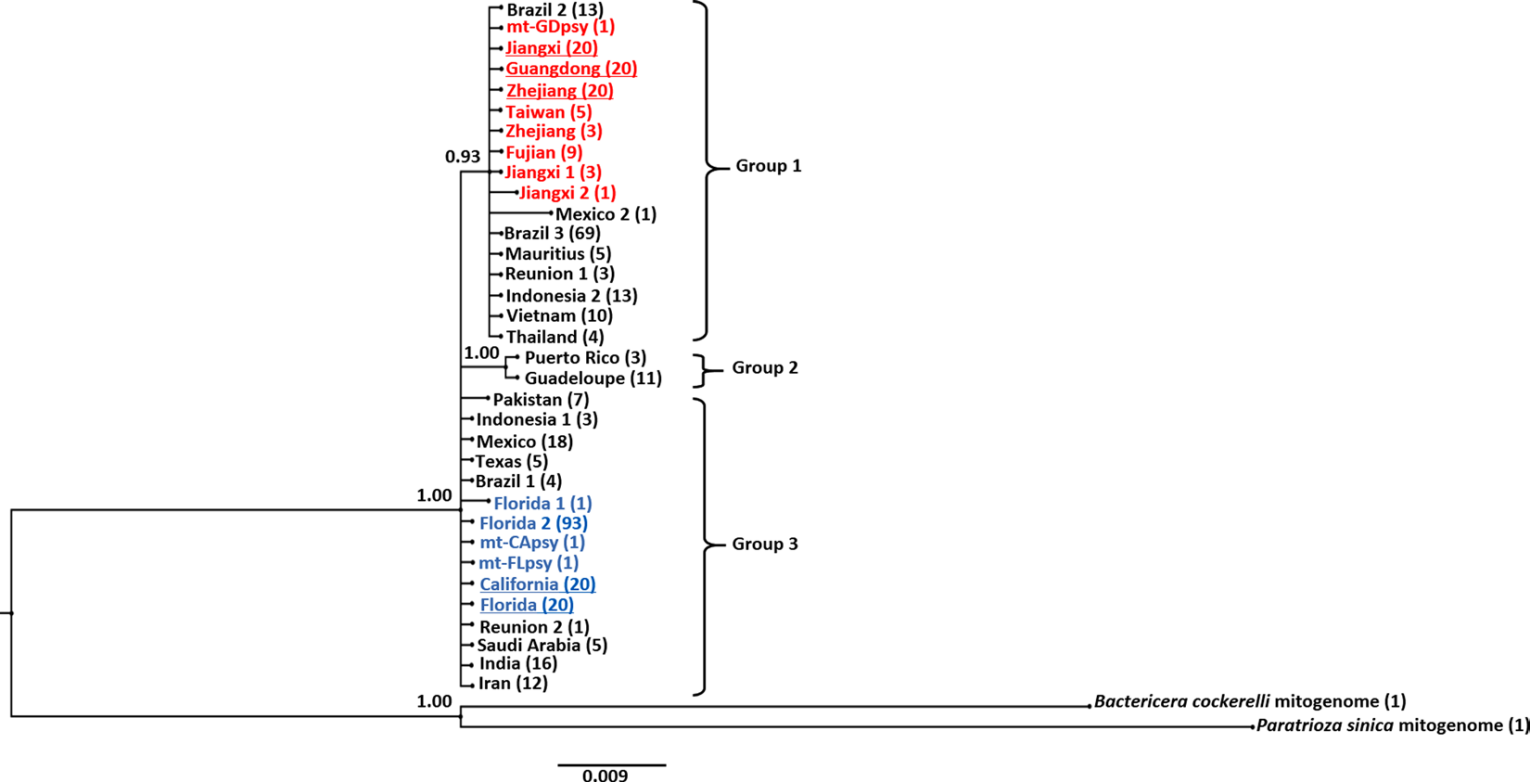
A Bayesian analysis of 318 *cox1* sequences (partial, 468 bp) of *Diaphorina citri* published in GenBank database (see Supplementary Table S3). Phylogenetic analysis was built by MrBayes v3.2, and numbers at the nodes are posterior probabilities of Bayesian inference method. Samples used in this study were underlined. Sequences from the USA were highlighted “blue” and sequences from China were “red”. Numbers in the brackets are representative number of sequences.

The length of *cox1* of ACP was 1,530 bp. There were 363 partial *cox1* gene sequences deposit in GenBank database (before July, 2016). Length of these sequences ranged from 433 to 996 bp. A 468 bp region was shared from 318 psyllids (see Supplementary Table S3) and they were used for phylogenetic tree construction, yielding three major *cox1* groups (Fig. 5). The 122 USA sequences including those of California and Florida were in Group 3. All Chinese sequences, including samples collected from three Provinces (Guangdong, Jiangxi and Zhejiang) were in Group 1. ACPs from Reunion, Brazil, Indonesia and Mexico distributed in two different groups (Group 1 and Group 3), but 94.7% (18/19) of ACPs from Mexico were belonged to Group 3 (Fig. 5).

### tRNA gene variations

Twenty of the 22 rRNA genes from both mt-CApsy and mt-FLpsy and mt-GDpsy (NC_030214) had standard cloverleaf structure, a typical feature of metazoan mitogenomes^27^. *trnSer* ^AGN^ could not form a stem-loop structure in the dihydrouridine arm (see Supplementary Fig. S1), the same as that of the reported Psylloidea mitogenomes^21, 23–26^. *trnTrp* did not have variable loop which was unique to ACP mitogenome (Fig. S1)^21^.

**Figure 6:**
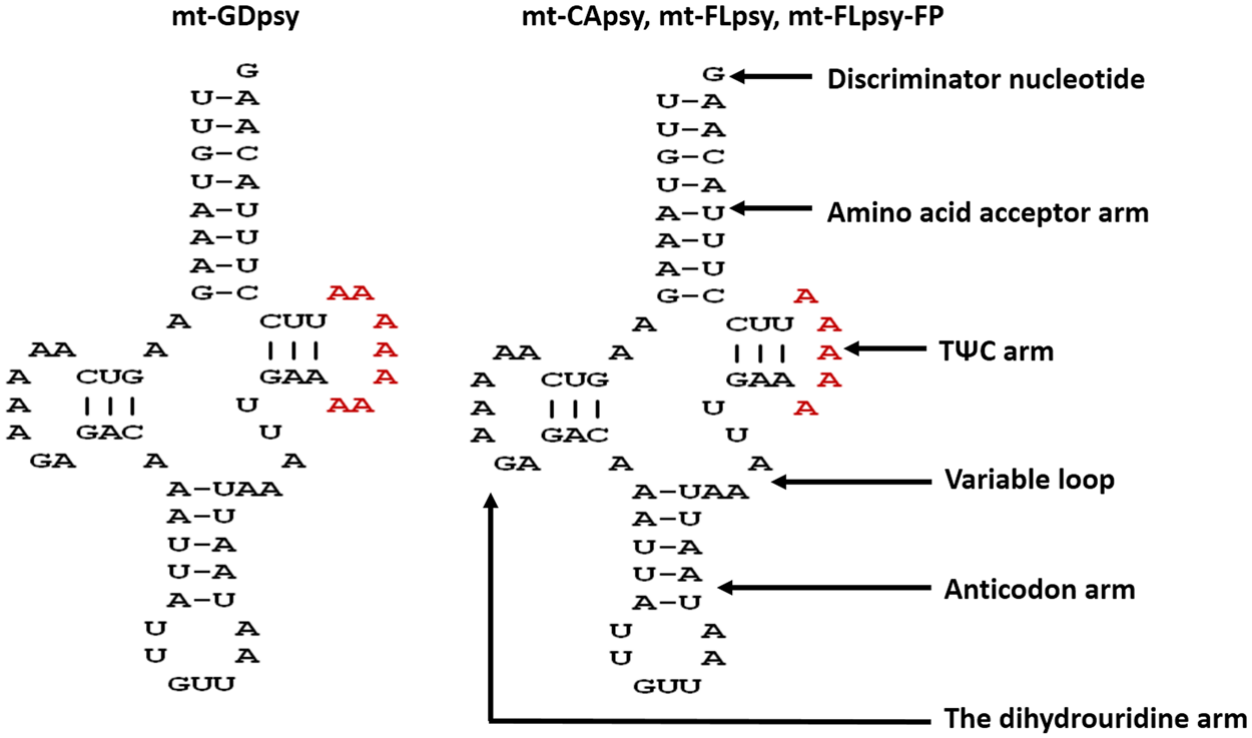
Secondary structures of *trnAsn* identified in the mitogenome of *Diaphorina citri*. Bar “-”, Watson-Crick base pairing. Bases highlighted in red indicated the different structure among mt-GDpsy (NC_030214), CApsy (KY426014), mt-FLpsy (KY426015) and mt-FLpsy-FP (NW_007378019).

In *trnAsn*, size of TΨC arm was different between mt-CApsy and mt-GDpsy (Fig. 6). mt-CApsy had two nucleotides (“AA”) deleted as comparing to mt-GDpsy. This was further confirmed by examining 100 additional psyllid samples from China and the USA using conventional PCR and Sanger sequencing (see Supplementary Table S1).

### PCR differentiation of ACPs between America and China at *nad1*-*nad5*-*nad4*

**Figure 7:**
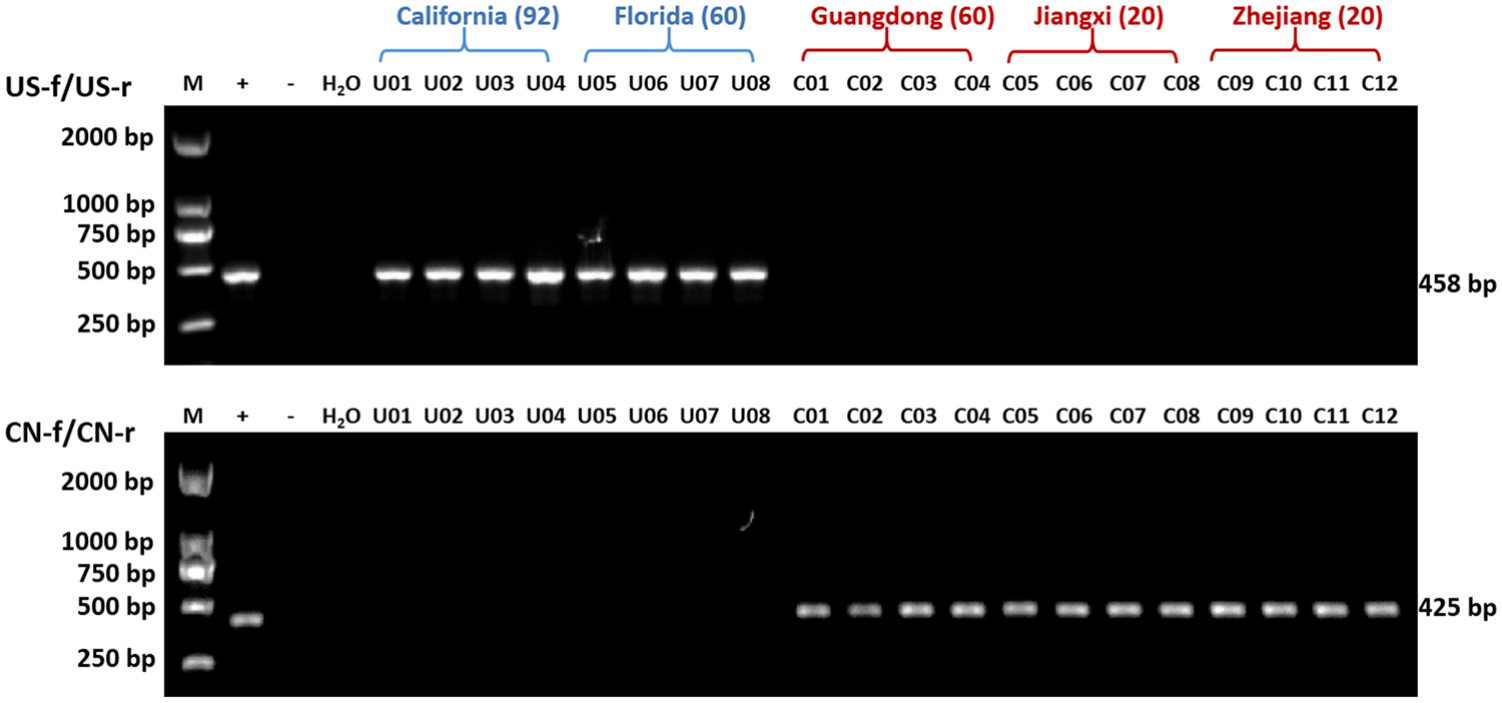
Differentiation of *Diaphorina citri* populations between China and USA based on primer sets US-f/US-r, and CN-f/CN-r. The samples from USA were highlighted by “blue” and China by “red”. Numbers in the brackets are representative number of sequences.

Based on SNP comparison between American and Chinese ACP mitogenome sequences used in this study, primer set US-f/US-r was designed at *nad1* and primer set CN-f/CN-r was designed in *nad5-nad4*. All 152 ACP samples from the USA (see Supplementary Table S1) were detected by primer set US-f/US-r with the presence of a 458 bp amplicon, but not by primer set CN-f/CN-r. Primer set CN-f/CN-r amplified a 425 bp amplicon from 100 ACP collected from three provinces in China (see Supplementary Table S1), but not those from USA as representatively shown in Fig. 7.

## Discussion

Historically, sequence analyses of a single mitochondrial gene *cox1* has played a major role in ACP population research^16–20^. In this study, efforts were made to obtain complete ACP mitogenome sequences from California and Florida to complement existing findings. Based on the analysis of all currently available psyllid mitogenome sequences, a monophyletic lineage of ACP among members of psyllids (Psylloidea) was established and all ACPs formed a unique cluster within Psylloidea Superfamily (Fig. 3). Furthermore, within ACPs, divergence of mt-GDpsy was observed. The 100% matches of additional 10 nearly-complete mitogenome sequences from each region, suggesting that the single complete mitogenome sequences were representative for each region.

Analyses of *cox1* gene (Fig. 5) and *trnAsn* gene (Fig. 6) sequences further supported the phylogenetic conclusion from the whole mitogenome analyses that Californian ACP grouped with Floridian ACP, rather than to Guangdong ACP. For *cox1*, as shown in Fig. 5, all ACPs can be primarily separated into three groups. All Chinese ACPs used in this study are in Group 1, whereas all American ACPs are in Group 3. Due to the limited number of samples analyzed, we could not further discuss whether Group 2 represents a third distinct *cox1* gene cluster. Taken together the results from analyses of mitogenome sequences (34 ACPs), *cox1* (463 samples), *trnAsn* (100 samples), and *nad1-nad4*-*nad5* (252 samples) (see Supplementary Table S1), this study suggested the presence of two distinct clusters: the Chinese ACP cluster (CAC) and the American ACP cluster (AAC).

Unlike the Asiatic origin of CLas strains in California^14, 28^, Californian ACP was likely of America origin. Therefore, CLas and ACP might have been introduced in two separate events. Likely, ACP was introduced in California through ground transportation from southeastern USA or Mexico with infested plant material. However, mitogenome sequence analysis of Mexico ACP is currently lacking. The time line of ACP detections seem to suggest a scenario of ACP movement from southeastern USA. ACP was first reported in Florida in 1998^11^. It was ten years later (2008) when ACP was found in California^13^. Although not the objective of this study, the origin of ACP in Florida deserves future research. One scenario was that Floridian ACP came from South America through Central America and the Caribbean^4^. Based on analyses in a single *cox1* gene, Boykin *et al*. suggested ACPs between the USA (Florida) and Caribbean (Puerto Rico and Guadeloupe) belong to different haplotype^16^, as Group 2 in Fig. 5, and De León *et al*. also found two separate introductions or founding events of ACP occurred in the Americas (South America and North America)^20^. Future research should evaluate these two ACP populations (Group 2 and 3) with more genes along with more ACP samples from Caribbean islands.

The recognition of separate introduction routes of ACP and CLas in California could have implications on HLB regulatory strategies in California, in particular strategies aimed at prevent establishment of CLas. Currently, citrus trees are surveyed for disease symptoms and the presence of psyllids. ACP are collected and analyzed for the presence of CLas. If any psyllids test positive, the tree from which the psyllids were collected and adjacent trees are analyzed for CLas. When plant tissue is diagnosed as positive for CLas, the tree is eliminated. Relying solely on the presence of ACP runs the risk of missing CLas infected trees in areas that currently are free of ACP. Additionally, these results suggest that efforts to prevent ACP from arriving in USA ports have been quite successful, but movement of plant materials by individuals is of great concern and is likely how CLas entered California.

Whole mitogenome sequence analyses revealed multiple amplicons were generated with the CR primer set (Fig. 2). Different primer sets were designed and the same results were observed. In general, animal mitogenomes have only one CR that controls the transcription and/or replication^22, 29–31^. This was the case in our previous mitogenome study with potato psyllid^26^ (Fig. 2a). In ACP, multiple CRs could mean the presence of multiple circular forms of its mitogenomes. The biological reason for this is unclear, albeit multiple CR amplicons have also been discovered in other arthropods such as *Boophilus microplus* and *Thrips imagini* ^32, 33^.

For ACP, *cox1* is the most studied gene, probably because of the easy access of this gene by conserved PCR primers^16–20^. As shown in Fig. 4, five SNPs were found in *cox1* between CAC and AAC. More SNPs are found in *nad4* (6 SNPs) and *nad5* (7 SNPs), suggesting that the two genes have better potential for future population diversity research. In this study, two primer sets CN-f/CN-r and US-f/US-r were designed from the SNPs in *nad5*, *nad4* and *nad1*, which can separate CAC and AAC psyllids (Fig. 7). This primer set can be utilized in future ACP diversity studies. Interestingly, *rrnL* also has 6 SNPs, higher than that of *cox1*. Although psyllid population diversity has mostly focused on PCGs, rRNA genes have been used in evaluation of potato psyllid diversity^34^. The complete mitogenome sequences of ACP presented in this study provides feasible access to every mitochondrial gene.

## Conclusion

In the present study, the complete mitogenome sequences of ACPs from California and Florida were acquired and compared. The Californian ACP was found to group with Floridian ACP, rather than ACP from three provinces in China. Further validations on three mitogenomic loci (*cox1* with 100 ACPs in this study and 318 ACPs from previous publications, *trnAsn* with 100 ACPs and *nad1-nad4-nad5* with 252 ACPs) supported the result of mitogenome sequence analysis. From these data, we suggested that ACP in California was likely not introduced from China based on our current ACP collection, but from somewhere in America, such as southeastern USA, because of the cross-continent transportation of citrus materials. This information is of HLB regulatory importance. However, accurate determination of the origin of Californian ACP requires further research with more ACP samples collected from more geographical locations such as Mexico, more locations in China, and around the world. Mitogenome sequencing and analyses can contribute and facilitate future ACP population studies.

## Materials and Methods

### ACP collection and DNA preparation

For mitogenome sequencing, individual ACP samples were collected from California and Florida, USA. For the Californian sample, a CLas-infected ACP was collected in San Gabriel, California by the California Department of Food and Agriculture (CDFA) during a state-wide HLB survey in 2015. For the Floridian sample, a CLas-free ACP was collected from Immokalee, Florida in August 11, 2015. DNAs were extracted using the DNeasy blood and tissue kit (Qiagen, Valencia, CA).

For population analyses, three mitogenomic loci, *cox1, trnAsn*, and *nad1-nad5-nad4*, were selected. A total of 482 additional ACP individuals (see Supplementary Table S1) were collected during the years of 2015 and 2016 from Guangdong, Jiangxi and Zhejiang in China, California and Florida. DNA was extracted from individual ACP at South China Agricultural University (Chinese ACPs), CDFA (Californian ACPs), and USDA-ARS at Parlier, California (Florida ACPs received in alcohol preservation). Voucher specimens of ACP from China are preserved at Entomological Specimen Room of South China Agricultural University in Guangzhou, China, and available at request.

### Mitogenome sequencing by MiSeq, assembling and annotation

Individual ACP DNA was amplified through illustra GenomiPhi V2 DNA Amplification Kit (GE Healthcare Inc., Waukesha, WI, USA). The amplified DNA samples were sequenced using Illumina MiSeq format (Illumina, San Diego, CA). *De novo* assembly of ACP genome sequences was performed with CLC Genomics Workbench v.7.5 (CLC Bio, Denmark). Mitogenomic contigs were identified by standalone BLASTn v.2.2.30^35^ using the complete mitochondrial sequence of ACP from Guangzhou, China (NC_030214) as reference and extracted using a Perl script. Alternatively, ACP mitogenome sequences were obtained by mapping using Bowtie2 v.2.2.6^36^ based on reference ACP mitogenome sequences (mt-GDpsy).

Variations of CR of mitogenomes were analyzed by conventional PCR using CR-f/CR-r: CGC AAC TGC TGG CAC AAA AT/GTC CTT TTT CAG GCA TCG CTC as primer and amplified DNAs as templates with the following PCR amplification procedure: initial denaturation for 3 min at 95 °C, followed by 35 cycles of denaturation for 45 s at 95 °C, annealing for 30 s at 55 °C, elongation for 2 min 30 s at 72 °C, and a final extension step of 72 °C for 10 min. The amplified DNA fragments were cut by NucleoSpin® Gel and PCR Clean-up kit (QIAGEN, Valencia, USA). PCR products of the respective DNA fragments were cloned into pEASY cloning vectors. Recombinant plasmid DNAs were purified by a QIAprep Spin Miniprep Kit (Qiagen), and sequenced using ABI 3130 DNA sequencer (ABI, Foster, CA, USA).

Nucleotide coverages of mitogenome sequences were calculated. The mitogenome sequences were annotated by Open Reading Frame Finder^37^ available at the website of National Center for Biotechnology Information (NCBI) with the invertebrate mitochondrial genetic codons and MITOS v.806^38^. Gene boundaries were reexamined and manually adjusted. PCGs and tRNA and rRNA genes were aligned with mt-GDpsy using Clustal W as implemented in MEGA v.6^39^.

### Mitogenome sequencing by PCR and Sanger sequencing

Nineteen primer sets (see Supplementary Table S2) were designed by Primer3 v.0.4.0^40^ referenced to the alignment of mt-CApsy, mt-FLpsy and mt-GDpsy. PCR amplicon sizes of each primer set were about 1 kb, readily overlapped by Sanger’s sequencing from both ends. The costs of PCR-Sanger sequencing mitogenome were substantially lower than that of MiSeq.

### Comparison of mitogenome nucleotide sequences

The Guangdong mt-GDpsy (NC_030214) and the shotgun Floridian ACP genome sequence (PRJNA29447) were downloaded from the GenBank database. The mitogenome in the Floridian ACP sequence was identified by BLASTn using mt-FLpsy as query. A sequence fragment (14,094 bp) from one contig (NW_007378019, 169,790 bp) was identified as the ACP mitogenome and named as mt-FLpsy-FP. Together with mt-CApsy and mt-FLpsy, all mitogenome sequences were aligned using Clustal W. SNPs in mitogenome genes among mt-CApsy, mt-GDpsy, mt-FLpsy and mt-FLpsy-FP were identified. SNPs were confirmed using ACPs collected from China, California and Florida using conventional PCR [FL-1f/FL-1r (ACT CAT AGG CAA TTT AGA GCG A/CGG ATT GAG GAT TGT CTG ATT CC) and FL-2f/FL-2r (GCA AGT AAT GGT CTC TTA AAC CA/ATA ATT GGT CAA GGC GAA GG)], and Sanger sequencing.

### *cox1* gene analyses

The 363 published *Diaphorina citri* cytochrome oxidase (*cox1*) sequences from 16 countries/regions were downloaded from the GenBank nucleotide database (before July, 2016). Among them, 318 sequences overlapped a common region (468 bp) (from 2,112 to 2,579 bp in mt-CApsy). After removal of redundant sequences by filtering out the same sequences from the same places, 26 representative sequences were obtained for construction of a phylogenetic tree (see Supplementary Table S3). An additional 40 ACP samples collected from USA (California and Florida) and 60 from China (Guangdong, Jiangxi and Zhejiang) were also included (see Supplementary Table S1). The corresponding *cox1* regions of *Bactericera cockerelli* (NC_030055) and *Paratrioza sinica* (NC_024577) were used as an outgroup. MrBayes v.3.2.5^41^ for the BI method, was used to construct phylogenetic trees. Two sets of four chains were allowed to run simultaneously for 1,000,000 generations, with sampling every 100 generations. After discarding the first 25% samples as burn-in, Bayesian posterior probability values were calculated in a consensus tree. The result was showed using FigTree v.1.4.2.

### tRNA genes analyses

The tRNA genes of ACP mitogenome were predicted by their cloverleaf secondary structure using tRNAscan-SE v.1.21 and ARWEN v.1.2^42, 43^. The variations in all tRNA genes among mt-CApsy, mt-GDpsy, mt-FLpsy and mt-FLpsy-FP were detected by alignment using Clustal W. SNPs or gaps were identified and verified by additional samples collected from USA (California, Florida) and China (Guangdong, Jiangxi and Zhejiang) (see Supplementary Table S1). Conventional PCR and Sanger sequencing were performed if a discrepant locus was found.

### Analyses of mitogenomes

Representative mitogenome sequences of Psylloidea (*Paratrioza sinica*: NC_024577; *Bactericera cockerelli*: NC_030055; *Cacopsylla coccinea*: NC_027087; *Pachypsylla venusta*: NC_006157) and, as an outgroup, Aphidoidea (*Schizaphis graminum*: NC_006158; *Aphis gossypii*: NC_024581) (Fig. 3) were downloaded from the GenBank database^23–26, 44^. The mitogenomes of Aphidoidea were used as outgroup. Sequences of 37 genes were extracted based on annotations. All sequences were concatenated and aligned by Clustal X^45^ using default parameters. For a cross-checking purpose, two software programs, PHYML v.3.0^46^ for the ML method and MrBayes v.3.2.5^41^ for the BI method, were utilized to construct phylogenetic trees. The method of BI analyses was the same as the phylogenetic analyses of *cox1* introduced above. For ML analyses, the optimal substitution model obtained from jModelTest v.2.1.7^47^ was used. Nodal support among branches was evaluated by bootstrap analysis with 100 replicates^26^.

### Development of specific PCR primers

SNPs in two variable regions (USA and China) were used to design specific primer sets for ACP in California/Florida and Guangdong of China with the Primer 3 program. Specificity was achieved by placing SNPs at the 3’ end of each primer. Primer US-f/US-r (CAG AAA AAA GCA AGG ATA TT/TAG GGT GAA ATT GAC ATT TC) was designed from the SNPs in *nad5* and *nad4* genes between mt-CApsy and mt-GDpsy. Primer CN-f/CN-r (CAT TAA ACC CTG AAA CAA GT/TTG GGT TAC TCC TTT AAT TG) was designed from *nad1* gene. These primers were tested against 152 ACP samples collected from the USA and 100 ACP samples from China (see Supplementary Table S1). PCR amplification was: initial denaturation for 1 min at 96 °C, followed by 30 cycles of denaturation for 30 s at 96 °C, annealing for 30 s at 53 °C, elongation for 30 s at 72 °C, and a final extension step of 72 °C for 4 min.

## Electronic supplementary material

Supplementary table and figure
