# Supplementary material for: Analyses of Mitogenome Sequences Revealed that Asian Citrus Psyllids (Diaphorina citri) from California Were Related to Those from Florida
Source: Sci Rep. 2017 Aug 31;7:10154. doi: 10.1038/s41598-017-10713-3 (PMC5578989; doi:10.1038/s41598-017-10713-3)
Supplement: Supplementary file 1 — Supplementary table and figure [file 41598_2017_10713_MOESM1_ESM.pdf]

**Analyses of Mitogenome Sequences Revealed that Asian Citrus Psyllids (*Diaphorina citri*) from California Were Related to Those from Florida**

Fengnian Wu[1,3], Luci Kumagai[2], Yijing Cen[1], Jianchi Chen[3]*, Christopher M. Wallis[3], MaryLou Polek[4], Hongyan Jiang[1], Zheng Zheng[1,3], Guangwen Liang[1], Xiaoling Deng[1]*

[1] Guangdong Province Key Laboratory of Microbial Signals and Disease Control / Laboratory of Insect Ecology, College of Agriculture, South China Agricultural University, Guangzhou, Guangdong, China

[2] California Department of Food and Agriculture, Plant Pest Diagnostic Center, Sacramento, California, USA

[3] United States Department of Agriculture-Agricultural Research Service, San Joaquin Valley Agricultural Sciences Center, Parlier, California, USA

[4] National Clonal Germplasm Repository for Citrus and Dates, Riverside, California, USA

Correspondence and requests for materials should be addressed to X.D. (email: xldeng@scau.edu.cn) or J.C. (email: Jianchi.Chen@ars.usda.gov)

**Supplementary Table S1. Numbers of *Diaphorina citri* samples used in this study.**

| Geographical origin | Whole mitogenome sequences [a] | Nearly complete Mitogenome sequences [b] | Selected mitogenomic loci [c] | | |
|---|---|---|---|---|---|
| | | | *cox1* | *trnAsn* | *nad1-nad4-nad5* |
| USA | | | | | |
| California | 1 (mt-CApsy, KY426014) | 10 | 20 | 20 | 92 |
| Florida | 2 (mt-FLpsy, KY426015; mt-FLpsy-FP, NW_007378019) | 10 | 20 | 20 | 60 |
| China | | | | | |
| Guangdong | 1 (mt-GDpsy, NC_030214) | 10 | 20 | 20 | 60 |
| Jiangxi | - | - | 20 | 20 | 20 |
| Zhejiang | - | - | 20 | 20 | 20 |
| Others[d] | - | - | 318 | - | - |
| Total= | 4 | 30 | 418 | 100 | 252 |

[a] Names and GenBank accession numbers of mitogenomes are in parentheses. mt-CApsy and mt-FLpsy were acquired through by Illumina MiSeq method. mt-FLpsy-FP and mt-GDpsy were not from this study.

[b] Sequences were acquired by PCR and Sanger sequencing method using 19 primer sets covering all mitogenome but the control region (CR). All sequence lengths were 14,094 bp.

[c] *cox1*=cytochrome oxidase *c* subunit 1, *trnAsn*=tRNA-Asn, *nad*=NADH dehydrogenase subunits.

[d] Source: GenBank database (before July, 2016).

## **Supplementary Table S2. PCR primers used for amplification of mitogenome of *Diaphorina citri* (Asian citrus psyllid, ACP).**

| Primers | Gene name of primers located [a] | Region [b] | F (5'-3') | R (5'-3') | Predicted PCR product size [c] |
|---|---|---|---|---|---|
| ACP-mt-1 | *nad2-cox1* | 501-1,482 | TGGCTCCCCTCCATATTTGA | AGATTGTCTTAATTCCAAACGGA | 982 |
| ACP-mt-2 | Y-*cox1* | 1,319-2,278 | AGAATTTACAGTTCCTCGCCT | TGGTTGCTGAAGTAAAATAAGCT | 960 |
| ACP-mt-3 | *cox1-cox2* | 2,019-2,996 | AGGAGGTGGAGACCCAATCT | TAGGCGAGGCTCTATCAAAA | 978 |
| ACP-mt-4 | *cox1-atp8* | 2,826-3,783 | ATGAATGCAAAACTCTCCCC | AAATAGTTAATCACGGAAGAGGT | 958 |
| ACP-mt-5 | K-*cox3* | 3,635-4,619 | GCAAGTAATGGTCTCTTAAACCA | ATAATTGGTCAAGGCGAAGG | 985 |
| ACP-mt-6 | *atp6*-G | 4,404-5,414 | TCCTTATAGCTCTCCTAGGAAAT | CAAAGGATCTATTAGTTGGAAGC | 1,011 |
| ACP-mt-7 | *cox3-nad5* | 5,208-6,139 | CACTCAAGTTCTCATGCGACTAA | TCTGTCGTGTTTTCAAGTAGT | 930 |
| ACP-mt-8 | G-*nad5* | 6,030-6,857 | ACTTAAAACACTGCCCTTTCA | ACTGTTTATATGTGCTGGGTT | 828 |
| ACP-mt-9 | *nad5-nad5* | 6,657-7,522 | ACACCTCCGTATCTACATCTTGT | TCAAAGATTTATATGGGCAAGGA | 866 |
| ACP-mt-10 | *nad5-nad4* | 7,409-8,377 | GCCTAGTAAAACTCCAACAATGC | TTTAGGTGGGTTAATTTTGACTG | 969 |
| ACP-mt-11 | *nad4-nad4L* | 8,096-9,334 | TCACCAAAGTCTTCTGATAG | GCATTTGTTAGTATTTATTAGATG | 1,239 |
| ACP-mt-12 | *nad4L-nad6* | 9,065-9,927 | GTCTCTTCGCATAGTCTCTACCA | GGGCCTTTGGTCAGAGTTATTA | 863 |
| ACP-mt-13 | *nad6-cob* | 9,758-10,744 | ACTCCCACTCAGTTATCAATATC | GAGGAGTATTTAGAGGGTTGGC | 987 |
| ACP-mt-14 | *cob-nad1* | 10,602-11,504 | CCCCTTTCACCCTTATTTTATCA | TCTTGCAGAACTTAATCGTACT | 903 |
| ACP-mt-15 | *nad1-rrnL* | 11,419-12,261 | ACCCGCCGTACTCAACATTA | GATTTGAGTTCAGACCGGCG | 843 |
| ACP-mt-16 | *nad1-rrnL* | 12,004-12,859 | GCAATGTTAGAAAAGCTACACT | TTAAGGGGAGGTAAAGTTGTTT | 856 |
| ACP-mt-17 | *rrnL-rrnS* | 12,675-13,563 | ACTGCAGCTATTTACATCAGCA | TTTATGTTAGGTCAAGGTGCA | 891 |
| ACP-mt-18 | *rrnS*-CR | 13,333-14,178 | ACCCAAATCCAGACACACCT | AGTCATAAGTCAATGGAGTCTCT | 846 |
| ACP-mt-19 | I-*nad2* | 14,983-693 | ACTCATAGGCAATTTAGAGCGA | CGGATTGAGGATTGTCTGATTCC | 700 |

[a] Transfer RNA genes are indicated by the single letter IUPAC-IUB abbreviations for their corresponding amino acid. Gene name before and after "-" represent the amplify range by the specific primers. Abbreviations: *atp6*, *atp8*, ATP synthase subunits 6 and 8 genes; *cob*, cytochrome oxidase *b* gene; *cox1-cox3*: cytochrome oxidase *c* subunit 1-3 genes; *nad1-6*, *nad4L*, NADH dehydrogenase subunits 1-6 and 4L; *rrnS*, *rrnL*, small and large ribosomal RNA subunits; CR, control region.

[b] Region used in this study was represented by mt-GDpsy (Accession: NC_030214).

[c] The PCR product size was predicted according to mt-GDpsy.

**Supplementary Table S3. Accession and locality of representative mitochondrial *cox1* from GenBank.**

| Regions | Accession No. | Representative numbers | References |
|---|---|---|---|
| Florida 1 | FJ190248 | 1 | Boykin et al. (2012) |
| Florida 2 | FJ190167 | 93 | Boykin et al. (2012) |
| Texas | FJ190177 | 5 | Boykin et al. (2012) |
| Brazil 1 | FJ190321 | 4 | Boykin et al. (2012) |
| Brazil 2 | FJ190228 | 13 | Boykin et al. (2012) |
| Brazil 3 | KC354739 | 69 | Guidolin et al. (2013; 2014) |
| Puerto Rico | FJ190260 | 3 | Boykin et al. (2012) |
| Indonesia 1 | FJ190336 | 3 | Boykin et al. (2012) |
| Indonesia 2 | FJ190263 | 13 | Boykin et al. (2012) |
| Vietnam | FJ190272 | 10 | Boykin et al. (2012) |
| Taiwan | FJ190283 | 5 | Boykin et al. (2012) |
| Pakistan | FJ190288 | 7 | Boykin et al. (2012) |
| Thailand | FJ190293 | 4 | Boykin et al. (2012) |
| Zhejiang | FJ190297 | 3 | Boykin et al. (2012) |
| Fujian | FJ190357 | 9 | Boykin et al. (2012) |
| Jiangxi 1 | FJ190366 | 3 | Boykin et al. (2012) |
| Jiangxi 2 | FJ190367 | 1 | Boykin et al. (2012) |
| Mexico 1 | FJ190300 | 18 | Boykin et al. (2012) |
| Mexico 2 | KJ453891 | 1 | Unpublished |
| Mauritius | FJ190312 | 5 | Boykin et al. (2012) |
| Reunion 1 | FJ190317 | 3 | Boykin et al. (2012) |
| Reunion 2 | FJ190318 | 1 | Boykin et al. (2012) |
| Saudi Arabia | FJ190337 | 5 | Boykin et al. (2012) |
| India | FJ190342 | 16 | Boykin et al. (2012) |
| Guadeloupe | FJ190346 | 11 | Boykin et al. (2012) |
| Iran | KC509572 | 12 | Lashkari et al. (2013) |

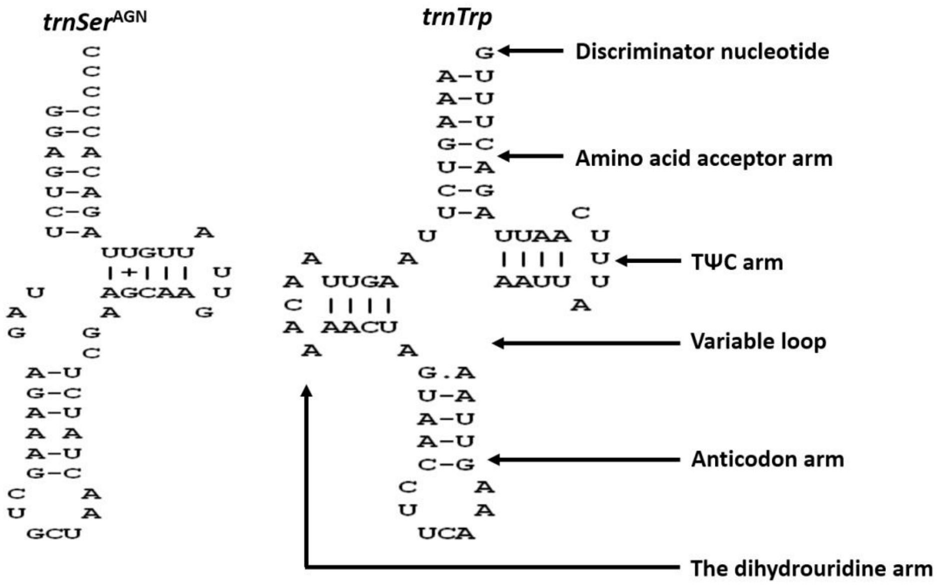


**Supplementary Figure S1. Secondary structures of tRNA with special structure identified in the mitogenome of Asian citrus psyllid.** Bar "-", Watson-Crick base pairing. Others are canonical base parings in tRNA: Plus sign "+", a paring between G and U; Dot "•" A paring between A and G.
